# Wigner’s Friend Scenarios and the Internal Consistency of Standard Quantum Mechanics

**DOI:** 10.3390/e23091186

**Published:** 2021-09-09

**Authors:** Dmitri Sokolovski, Alexandre Matzkin

**Affiliations:** 1 Departmento de Química-Física, Universidad del País Vasco, UPV/EHU, 48940 Leioa, Spain; 2 IKERBASQUE, Basque Foundation for Science, 48011 Bilbao, Spain; 3 Laboratoire de Physique Théorique et Modélisation, CNRS Unité 8089, CY Cergy Paris Université, CEDEX, 95302 Cergy-Pontoise, France; alexandre.matzkin@u-cergy.fr

**Keywords:** quantum mechanics, quantum measurements, quantum interference, Feynman’s paths, Wigner’s friend problem

## Abstract

Wigner’s friend scenarios involve an Observer, or Observers, measuring a Friend, or Friends, who themselves make quantum measurements. In recent discussions, it has been suggested that quantum mechanics may not always be able to provide a consistent account of a situation involving two Observers and two Friends. We investigate this problem by invoking the basic rules of quantum mechanics as outlined by Feynman in the well-known “*Feynman Lectures on Physics*”. We show here that these “Feynman rules” constrain the a priori assumptions which can be made in generalised Wigner’s friend scenarios, because the existence of the probabilities of interest ultimately depends on the availability of physical evidence (material records) of the system’s past. With these constraints obeyed, a non-ambiguous and consistent account of all measurement outcomes is obtained for all agents, taking part in various Wigner’s Friend scenarios.

## 1. Introduction

The celebrated Wigner’s Friend thought experiment [1] sought to expose the possible ambiguities of the quantum theory when it is applied to measurements determined by intelligent agents. In this thought experiment, an Observer is requested to measure a sealed laboratory in which a Friend is making a measurement. As Wigner argued, if the laboratory contained only an atom going through the inhomogeneous magnetic field of a Stern–Gerlach device, the laboratory’s evolution would be unitary. However, if a Friend is inside and actually measures the spin, should the Observer, who assumes quantum theory applies to systems of any scale, still describe the situation by unitary evolution?

Recently, an extended version of this Wigner’s Friend setup was proposed [2,3]. This extended version involves two external Observers each measuring a different laboratory, containing a Friend who is measuring a spin. According to Frauchiger and Renner [2], whose setup employed non-maximally entangled spin states, quantum mechanics becomes inconsistent in the following sense. An inference based on the Friends’ observed outcomes implies that the external Observers should never obtain a particular set of outcomes since the corresponding probability will vanish. However, a direct computation which ignores the Friends’ outcomes indicates that the same probability *is not* zero. In [3], Brukner, who employed maximally entangled spin states, concluded that the facts (as defined by the agents’ measurements) cannot be the same for each Observer and their associated Friend. This conclusion is reached by setting up an inequality involving correlations between the Friends’ and the external Observers’ outcomes, such that it may be obeyed only if these outcomes are observer-independent. It is then shown that the inequality is violated if quantum mechanics is applied.

Several authors have scrutinised both of the results reported in Refs. [2,3] and the implicit or explicit assumptions made in deriving them. Some of these works analysed the extended Wigner’s friend setup within frameworks arising in specific interpretations of quantum mechanics, in which the prescriptions for computing the probabilities of the measured outcomes are different from those followed by Frauchiger and Renner, or by Brukner. Such studies employed different variants of the de Broglie–Bohm interpretation [4,5], the consistent histories formalism [6], or the decoherence approach [7,8]. The Bayesian and relational approaches to quantum mechanics were used for the same purpose in [9,10]. Yet more elaborate methods included deriving theory-independent inequalities assuming a universal validity of all events to all agents [11], or adding non-invasive weak measurements in order to allow the external Observers to monitor the state of the Friends’ laboratories [12]. Translating the Wigner–Friend setup into an interferometric setup where the external Observers actions may erase a photon’s memory state was considered in [13]. A classical analogy of the scenario was proposed and studied in [14].

Among the viewpoints, thus, expressed, still missing is a standard textbook account of what happens in the extended Wigner–Friend scenarios. Arguably, the approach pioneered by Feynman in his texts [15,16] is the most basic pragmatic implementation of the standard quantum theory. Feynman gives a set of rules stating how probabilities should be computed from the amplitudes, viz. through a “sum over virtual paths” (involving amplitudes) or a “sum over real paths” (involving probabilities). Recently [17], we applied Feynman’s rules for adding the probability amplitudes to the original Wigner–Friend experiment. In [17], a coherent narrative was constructed by taking into account stable material records produced by each act of observation. In this paper, we extend the approach to more complex scenarios, in order to look for inconsistencies, similar to those discussed in [2,3,4,5,6,7,8,9,10,11,12,13]. We will ask whether similar problems would also arise in the frame work proposed in [15,16] and, if they would, then in what particular form? We will see that the rules, given by Feynman in [15], limit the a priori assumptions which can be made about Wigner–Friend scenarios, since some assumptions will violate these rules. This is because the existence of the probabilities of measurement outcomes relies on the existence of the corresponding material records.

The rest of the paper is organised as follows. In Section 2, we summarise rules given by Feynman. In Section 3, we apply the rules to the original Wigner–Friend problem. Section 4 discusses a more complex scenario, involving a total of four Observers. In Section 5, we show how one can (although by no means needs to) arrive at a logical contradiction. In Section 6 we resolve the contradiction by a straightforward application of Feynman’s prescriptions [15,16]. Our conclusions are in Section 7. Additional relevant details will be found in several Appendices.

## 2. Feynman Rules

For the purpose of our discussion, the general rules of [15] (see also Ch. 1 of [16]) can be condensed into the following propositions.

(A) Quantum mechanics provides probabilities of the outcomes of a series of measurements accessible, at least in principle, to an Observer or Observers.
(B) Probability is computed as the absolute square of a complex valued probability amplitude, *at the end* of the experiment.
(C) If a route consists of several parts, the amplitudes of the parts must be multiplied.
(D) The amplitudes are added if several routes leading to the same outcome are not distinguished by an experiment, even *in principle.*
(E) The amplitudes are never added for different and distinct final states.
(F) The amplitudes are never added if information about the final state is in principle available, regardless of whether this information is known or used.

These principles are readily applied to an experiment consisting of several measurements, created one after another on the same quantum system. Note that when considering a series of outcomes, the resulting probabilities can be obtained with the help of amplitudes evaluated for all relevant virtual (Feynman) paths connecting the states in the Hilbert space of the measured system. Note also that different sets of measurements may lead to different (incompatible) statistical ensembles. The different steps required to implement these rules are recalled in Appendix A.

## 3. One Wigner and One Friend

In the original Wigner–Friend scenario (WFS), Wigner [1] considers an Observer, say *W*, observing a friend, *F*. Using a device *D*, *F* measures a two-level system, initially in a state $|s_{0}\rangle$, in a basis |up〉 and |down〉. The possible states of the system (*S*), device (*D*), and the memory (μ), therefore, are:

(1) |i〉⊗|D(i)〉⊗|μ(i)〉i=up,down

In [1], Wigner argued that a linear superposition

(2) $$\sum_{i}\langle i|s_{0}\rangle |i\rangle ⊗|D(i)\rangle ⊗|\mu (i)\rangle$$

could not represent F’s situation once their measurement is completed, since Equation (2) appears to imply an uncertainty regarding *F*’s outcome, resolved only after W measures the composite {*S* + *D* + μ}. The question of whether Equation (2) should be correct from W’s viewpoint seemed to remain open, assuming W is able to coherently control the composite.

A different account of Wigner’s experiment can be given with the help of Feynman rules of Section 2. As in the example in Appendix B, it is sufficient to consider virtual scenarios for the system, with an understanding [the rule (D) of Section 2] that their amplitudes, or probabilities must be added, depending on whether the scenarios can or cannot be distinguished. To distinguish between possible scenarios one would require a record of the system’s earlier condition (a memory, a note, or an identical copy of the system) to be available when the experiment is finished, and the results are obtained [cf. the rule (B)]. By the rule (D), it does not matter whether all the records were inspected by the Observer(s), since it is their mere existence which determines how the amplitudes should be handled. It is important to note that if *W*’s action succeeds in erasing all records made by *F* earlier, then, by the rule (D), all information about the state of the system at the time of *F*’s measurement should be irretrievably lost to interference.

Elaborating on Wigner’s original scenario, let us consider the following situations, similar to those discussed in detail in Appendix B.

(I) W measures only the system in a different orthogonal basis, |fail〉=α|up〉+β|down〉 and |ok〉=γ|up〉+δ|down〉, so *F*’s memory and device remain untouched. At the end of the experiment, i.e., just after *W*’s measurement, there are several possibilities of recovering *F*’s outcome. For example, W could (i) look at *F*’s probe, (ii) ask *F*, who will have to consult their memory, or (iii) ask *F* to look at their device again. The probabilities of the four possible outcomes are, therefore, given by:

(3) $$P^{\prime }\left(j,i\right)={\left|A^{S}\left(j\leftarrow i\leftarrow s_{0}\right)\right|}^{2},i=up,down,\;j=fail,ok.$$

and:

(4) $$P^{\prime }\left(j\right)=\sum_{i}P\left(j,i\right).$$

$A^{S}\left(j\leftarrow s_{0}\right)=\langle j\left|U\left(t,t_{0}\right)\right|s_{0}\rangle$ is generically the transition amplitude connecting an initial state $|s_{0}\rangle$ at time $t_{0}$ to a state |j〉 at time *t*, where *U* is the system evolution operator. Following the rule (C), the amplitude, if an intermediate state |i〉 is chosen, is given by $A^{S}\left(j\leftarrow i\leftarrow s_{0}\right)=\langle j\left|i\rangle \langle i\right|s_{0}\rangle$, where we assume that the system has no own dynamics. Note that by (F) of the previous Section, Equation (4) holds even if the inquiry about *F*’s outcome was never made; it suffices that it could be made *in principle*. Note also that *W* can also measure the joint systems {S+D}, {S+μ}, and {D+μ} in any orthogonal basis, e.g., |Fail〉=α|D(up)〉⊗|up〉+β|D(down)〉⊗|down〉 and |Ok〉=γ|D(up)〉⊗|up〉+δ|D(down)〉⊗|down〉, etc. Equations (3) and (4) would continue to be valid for as long as at least one record, carried by μ, *D*, or *S*, is available once the experiment is finished.

(II) Assume now that *W* decides to measure the composite {S+D+μ} in a basis.

(5) $$\begin{array}{c} |Fail\rangle =\alpha |\mu (up)\rangle ⊗|D(up)\rangle ⊗|up\rangle +\beta |\mu (down)\rangle ⊗|D(down)\rangle ⊗|down\rangle , \end{array}$$

$$\begin{array}{c} |Ok\rangle =\gamma |\mu (up)\rangle ⊗|D(up)\rangle ⊗|up\rangle +\delta |\mu (down)\rangle ⊗|D(down)\rangle ⊗|down\rangle \;. \end{array}$$

In this situation, all records of *F*’s outcome would be erased, and the system’s virtual paths $\left\{j\leftarrow up\leftarrow s_{0}\right\}$ and $\left\{j\leftarrow down\leftarrow s_{0}\right\}$, j=fail,ok could not be distinguished. The only outcomes are those obtained by *W* and, in accordance with the rule (D) of the previous Section, we obtain:

(6) $$P^{\prime \prime }\left(j\right)={\left|A^{S}\left(j\leftarrow up\leftarrow s_{0}\right)+A^{S}\left(j\leftarrow down\leftarrow s_{0}\right)\right|}^{2},j=Fail,Ok.$$

In summary, if we define a Wigner’s Friend setup by the requirement that both F and W make full measurements with outcomes confirmed by material records at the end, then it is represented by the scenario (I). Case (II) is, by necessity, different since the records, duly produced by *F* earlier, did not survive until the end of the experiment. In both cases, the application of the Feynman rules given above led to an unambiguous answer. The probabilities $P^{\prime }\left(j\right)$ and $P^{\prime \prime }\left(j\right)$ of cases (I) and (II) are different, because they refer to different measurements conducted by *W*. Disagreements of this type will become more pronounced in a more complex extended Wigner–Friend scenario, which we discuss next.

## 4. Two Wigners and two Friends

In a more complex Wigner’s friend scenario (2W2FS), considered in [2], there are two two-level systems, called a “coin” and a “spin”, and four participants: a pair of Friends, $\bar{F}$ and *F*, and two external Observers, $\bar{W}$ and *W*. $\bar{F}$ measures the coin (C) and *F* measures the spin (S), each in their own basis. The orthonormal measurement basis of the coin consists of states |heads〉 and |tails〉. The spin states in *F*’s measurement basis will be labeled |up〉 and |down〉. The Friends “laboratories” (*L*, $\bar{L}$) consist of all material devices and objects, involved in the measurements (*D*, $\bar{D}$), (initially in states |D(0)〉 and $|\bar{D}\left(0\right)\rangle$) plus the coin and the spin, respectively. In order to simplify the already cumbersome notations, we included *F* and $\bar{F}$’s memories into what we now call *D* and $\bar{D}$ (note that strictly speaking, we only need the Friends’ memories or records to be inside their respective laboratories as this is what quantum mechanics applies to; this can be rendered metaphorically as *F* and $\bar{F}$ and *F* being inside *L* and $\bar{L}$). A measurement entangles the contents of a lab with the coin or the spin according to:

(7) $$\begin{array}{c} |\bar{D}\left(0\right)\rangle ⊗|\phi^{C}\rangle \to \langle heads|\phi^{C}\rangle |\bar{D}\left(heads\right)\rangle ⊗|heads\rangle +\langle tails|\phi^{C}\rangle \left|\bar{D}\left(tails\right)\rangle ⊗\right|tails\rangle \end{array}$$

$$\begin{array}{c} \equiv \langle heads|\phi^{C}\rangle |\bar{L}\left(heads\right)\rangle +\langle tails\left|\phi^{C}\rangle \right|\bar{L}\left(tails\right)\rangle ,\\ \left|D\left(0\right)\rangle ⊗\right|\phi^{S}\rangle \to \langle up|\phi^{S}\rangle |D\left(up\right)\rangle ⊗|up\rangle +\langle down|\phi^{S}\rangle \left|\bar{D}\left(down\right)\rangle ⊗\right|down\rangle \\ \equiv \langle heads|\phi^{S}\rangle |L\left(up\right)\rangle +\langle down|\phi^{C}\rangle |L\left(down\right)\rangle , \end{array}$$

where $|\phi^{C}\rangle$ and $|\phi^{S}\rangle$ are arbitrary states of the coin and the spin, and $|\bar{L}\left(has/tails\right)\rangle$ and |L(up/down)〉 are the states of the entire laboratories.

The development is as follows: Neither the coin nor spin have their own dynamics. At $t=t_{0}$ the coin, the spin, and the two labs are prepared in a state:

(8) $$|\Psi_{0}\rangle =[|heads\rangle +\sqrt{2}\left|tails\rangle \right]/\sqrt{3}⊗|down\rangle ⊗|\bar{D}\left(0\right)\rangle ⊗|D\left(0\right)\rangle .$$

Then, at $t_{1}>t_{0}$, $\bar{F}$’s device is coupled to the coin according to (7). At a $\tau >t_{1}$, the spin and coin are coupled by the means of a Hamiltonian ${\hat{H}}_{int}=i\left(\pi /4\right)|tails\rangle \langle tails|⊗\left[|up\rangle \langle down\right|-|down\rangle \langle up|]\delta \left(t-\tau +\epsilon /2\right)$, so that the corresponding evolution operator is:

(9) $$\begin{array}{c} {\hat{U}}^{c+s}\left(\tau ,\tau -\epsilon \right)[|heads\rangle +\sqrt{2}\left|tails\rangle \right]/\sqrt{3}⊗|down\rangle \;\;\;\;\;\;\;\;\;\;\;\;\;\;\; \end{array}$$

$$\begin{array}{c} =\left|heads\rangle \langle heads\right|⊗{\hat{1}}_{spin}+|tails\rangle \langle tails|⊗[{\hat{1}}_{spin}+\left|up\rangle \langle down\right|-\left|down\rangle \langle up|\right]/\sqrt{2}.\; \end{array}$$

One can say that $\bar{F}$ sends *F* the spin in a state |down〉 if their outcome is Heads, or in a superposition $(|up\rangle +\left|down\rangle \right)/\sqrt{2}$, if it is Tails. At $t_{2}>\tau$, *F*’s device is coupled to the spin according to (7).

Finally, at a $t_{3}>t_{2}$, $\bar{W}$ and *W* measure $\bar{F}$’s and *F*’s entire laboratories in the bases:

(10) $$\begin{array}{c} |\bar{ok}\rangle =\left[\right|\bar{L}\left(heads\right)\rangle -\left|\bar{L}\left(tails\right)\rangle \right]/\sqrt{2}, \end{array}$$

and:

(11) $$\begin{array}{c} |fail\rangle =[|L\left(up\right)\rangle +|L\left(down\right)\rangle ]/\sqrt{2},\;|ok\rangle =[|L\left(up\right)\rangle -|L\left(down\right)\rangle ]/\sqrt{2}, \end{array}$$

using couplings similar to those in Equation (7). 

## 5. A Contradiction

The apparent contradiction associated with the scenario of the previous Section, put forward in [2], can be summed up in the following manner [12]. By $t=t_{3}$, a unitary evolution, including the couplings (7) and (9) converts the initial state (8) into:

(12) $$|\Phi \left(t_{3}\right)\rangle =\left[\right|\bar{L}\left(heads\right)\rangle ⊗|L\left(down\right)\rangle +|\bar{L}\left(tails\right)\rangle ⊗|L\left(up\right)\rangle +|\bar{L}\left(tails\right)\rangle ⊗\left|L\left(down\right)\rangle \right]/\sqrt{3}.$$

It does not contain a term $|\bar{L}\left(heads\right)\rangle ⊗|L\left(down\right)\rangle$, so the likelihood of $\bar{F}$ and *F* jointly obtaining the outcomes Heads and Up is zero:

(13) $$\begin{array}{c} P(Heads,Up)=0, \end{array}$$

while, obviously:

(14) $$\begin{array}{c} P(Heads,Down)=P(Tails,Down)=P(Tails,Up)=1/3. \end{array}$$

However, in another equivalent representation, the state (12) misses the term |ok〉⊗|tails〉:

(15) $$|\Phi \left(t_{3}\right)\rangle =\left[\right|\bar{L}\left(heads\right)\rangle ⊗|fail\rangle -|\bar{L}\left(heads\right)\rangle ⊗|ok\rangle +2|\bar{L}\left(tails\right)\rangle ⊗\left|fail\rangle \right]/\sqrt{6},$$

so that:

(16) $$\begin{array}{c} P(Tails,Ok)=0. \end{array}$$

In yet another representation, one has:

(17) $$|\Phi \left(t_{3}\right)\rangle =\left[2|down\rangle ⊗\right|\bar{fail}\rangle +|L\left(up\right)\rangle ⊗|\bar{fail}\rangle +2|L\left(up\right)\rangle ⊗\left|\bar{ok}\rangle \right]/\sqrt{6},$$

and:

(18) $$\begin{array}{c} P(Down,\bar{Ok})=0. \end{array}$$

Finally, the direct calculation of a probability $P(Ok,\bar{Ok})$ yields:

(19) $$\begin{array}{c} P\left(Ok,\bar{Ok}\right)=|\langle ok|\langle \bar{ok}{\left|\Phi \left(t_{3}\right)\rangle \right|}^{2}=1/12. \end{array}$$

Now, the following reasoning leads to a contradiction,

(i) By Equation (18), ($\bar{W}$’s) result “$\bar{Ok}$” implies an “Up” result for *F*’s spin.
(ii) By Equation (16) (*W*’s) result “Ok” implies a “Heads” result for $\bar{F}$’s.
(iii) However, by Equation (13) an “Up” result for the spin implies a “Tails” result for the coin; however, that is not true according to Equation (19).

Hence,

(iv) $\bar{W}$ and *W* will never obtain their “$\bar{Ok}$” and “Ok” results at the same time.

However, Equation (19) shows that conclusion (iv) is wrong.

One way to deal with the contradiction is to treat all apparently legitimate results (13), (16), and (18) as the participants’ personal experiences (observer-dependent facts [3]) which should not be compared against each other.

Another possibility is to select the right answer for the 2W2F scenario of Section 4 in terms of the available records, and find out which scenarios can be attached to the statements (i)–(iii).

Next, we will do so with the help of the Feynman’s rules of Section 2

## 6. An Analysis

Our analysis of the 2W2FS of Section 4 will be similar to that of the WFS Section 3. As discussed in Section 2, the Feynman rules rely on the existence material records for measurement outcomes. If all records are retained, the rules (E) and (F) imply a sum of probabilities over the “real” paths followed by the system. In the absence of such records, the rule (D) of Section 2 prescribes a sum of probability amplitudes over the system’s virtual paths. In order to clarify the origin of the conflicting statements (25), (28), and (32) at the heart of the contradiction in Section 5, we consider several cases where $\bar{W}$ or *W* either leave $\bar{F}$’s or *F*’s records untouched, or choose to erase these records. In particular, if $\bar{W}$ chooses to measure only the coin, we can still use Equation (11), provided we redefine the states |fail〉 and |ok〉 by replacing L(up/down)〉=|D(up/down)〉⊗|up/down〉 with:

(20) $$\begin{array}{c} |L(up)\rangle \equiv |up\rangle ,\;|L(down)\rangle \equiv |down\rangle ,\; \end{array}$$

It will be possible to deduce the coin’s condition at $t=t_{1}$ from the condition of $\bar{D}$ at $t=t_{3}$, and we will say that *F’s record is preserved*.

Similarly, $\bar{W}$ may choose to *preserve $\bar{F}$’s record*, by redefining the states $|\bar{L}\left(heads/tails\right)\rangle$ to be:

(21) $$\begin{array}{c} |\bar{L}\left(heads\right)\rangle \equiv \left|heads\rangle ,\;\right|\bar{L}\left(tails\right)\rangle \equiv |tails\rangle .\;\;\; \end{array}$$

As in Section 3, it is sufficient to consider the virtual scenarios in the four dimensional Hilbert space of the joint system {coin+spin}, a welcome specification, given the number of the variables involved.

### 6.1. The System’s Virtual Paths

**Figure 1:**
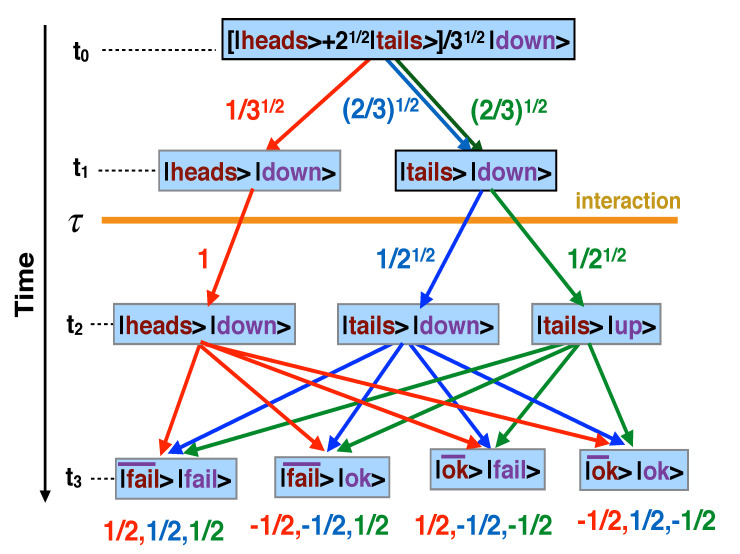
Virtual paths in the Hilbert space of the composite {coin+spin} (only the ones with non-zero amplitudes are shown). $\bar{F}$ measures the coin $t=t_{1}$, *F* does the same at $t=t_{2}$, and both $\bar{W}$ and *W* measure at $t=t_{3}$. The coupling (9) between the coin and the spin occurs at t=τ. The number next to segment of a path, or below it, is the value of the matrix element of the evolution operator. The full path amplitude is a product of all such numbers.

There are twelve virtual paths of the composite {coin+spin} with non-zero amplitudes. The probability amplitudes for each of the paths shown in Figure 1, are given by:

(22) $$\begin{array}{c} A_{1}\equiv A\left(\bar{fail}⊗fail\leftarrow heads⊗down\leftarrow heads⊗down\leftarrow \Phi_{0}\right)=1/\sqrt{12}, \end{array}$$

$$\begin{array}{c} A_{2}\equiv A\left(\bar{fail}⊗fail\leftarrow tails⊗down\leftarrow tails⊗down\leftarrow \Phi_{0}\right)=1/\sqrt{12},\\ A_{3}\equiv A\left(\bar{fail}⊗fail\leftarrow tails⊗up\leftarrow \;tails⊗down\leftarrow \Phi_{0}\right)=1/\sqrt{12},\\ A_{4}\equiv A\left(\bar{fail}⊗ok\leftarrow heads⊗down\leftarrow heads⊗down\leftarrow \Phi_{0}\right)=-1/\sqrt{12},\\ A_{5}\equiv A\left(\bar{fail}⊗ok\leftarrow tails⊗down\leftarrow tails⊗down\leftarrow \Phi_{0}\right)=-1/\sqrt{12},\\ A_{6}\equiv A\left(\bar{fail}⊗ok\leftarrow tails⊗up\leftarrow tails,down⊗\leftarrow \Phi_{0}\right)=1/\sqrt{12},\\ A_{7}\equiv A\left(\bar{ok}⊗fail\leftarrow heads⊗down\leftarrow heads⊗down\leftarrow \Phi_{0}\right)=1/\sqrt{12},\\ A_{8}\equiv A\left(\bar{ok}⊗fail\leftarrow tails⊗down\leftarrow tails⊗down\leftarrow \Phi_{0}\right)=-1/\sqrt{12},\\ A_{9}\equiv A\left(\bar{ok}⊗fail\leftarrow tails⊗up\leftarrow tails⊗down\leftarrow \Phi_{0}\right)=-1/\sqrt{12},\\ A_{10}\equiv A\left(\bar{ok}⊗ok\leftarrow heads⊗down\leftarrow heads⊗down\leftarrow \Phi_{0}\right)=-1/\sqrt{12},\\ A_{11}\equiv A\left(\bar{ok}⊗ok\leftarrow tails⊗down\leftarrow tails⊗down\leftarrow \Phi_{0}\right)=1/\sqrt{12},\\ A_{12}\equiv A\left(\bar{ok}⊗ok\leftarrow tails⊗up\leftarrow tails⊗down\leftarrow \Phi_{0}\right)=-1/\sqrt{12}, \end{array}$$

where $|\Phi_{0}\rangle =[|heads\rangle +\sqrt{2}\left|tails\rangle \right]/\sqrt{3}⊗|down\rangle$. The calculation of the probabilities for all choices performed by $\bar{W}$ and *W* is now reduced to a simple book-keeping exercise.

### 6.2. Both $\bar{F}$’s and F’s Records Are Erased by $\bar{W}$ and W

We begin with the scenario where $\bar{W}$ nor *W* choose the bases $\bar{L}\left(heads/tails\right)\rangle =\left|\bar{D}\left(heads/tails\right)\rangle ⊗\right|heads/tails\rangle$ and L(up/down)〉=|D(up/down)〉⊗|up/down〉 for their respective measurements. With the records of $\bar{F}$’s and *F*’s records destroyed, the situation is similar to the one discussed in Section 6.1 (cf. Equations (A7)–(A14)). In Figure 1, all paths leading to the same final state interfere, and following the rules of Section 2 and Appendix A, we sum the corresponding amplitudes to obtain:

(23) $$\begin{array}{c} P\left(\bar{Fail},Fail\right)={\left|A_{1}+A_{2}+A_{3}\right|}^{2}=9/12,\; \end{array}$$

$$\begin{array}{c} P\left(\bar{Fail},Ok\right)={\left|A_{4}+A_{5}+A_{6}\right|}^{2}=1/12,\;\\ P\left(\bar{Ok},Fail\right)={\left|A_{7}+A_{8}+A_{9}\right|}^{2}=1/12,\;\\ P\left(\bar{Ok},Ok\right)={\left|A_{10}+A_{11}+A_{12}\right|}^{2}=1/12.\; \end{array}$$

**Figure 2:**
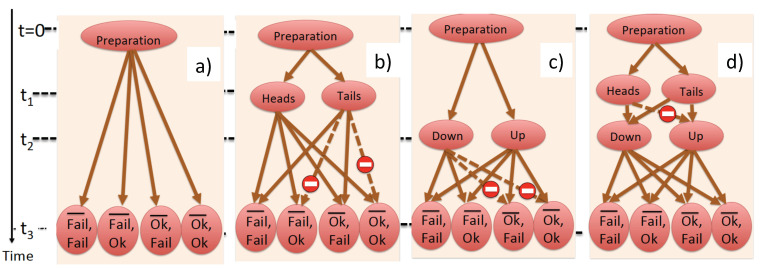
Real paths connecting the available outcomes if (**a**) both $\bar{F}$’s and *F*’s records are preserved; (**b**) only $\bar{F}$’s record is preserved; (**c**) only *F*’s record is preserved and (**d**) both $\bar{F}$’s and *F*’s records are destroyed. The probabilities for the paths shown by the dashed lines vanish.

The corresponding statistical ensemble, shown in Figure 2a, consists of four real paths connecting a successful preparation with $\bar{W}$’s and *W*’s outcomes and we recover Equation (19) as the correct result for $P(\bar{Ok},Ok)$.

Note that rule D) of Section 2 (which represents here the Uncertainty Principle [15]) forbids making any assumptions about whether the coin was “heads” or “tails” at $t=t_{1}$, or if the spin was “up” or “down at $t=t_{2}$. Note also that one can still inspect, for example, the state of $\bar{F}$’s record after $\bar{W}$ has completed their measurement. However, just as in the example at the end of Appendix B, finding it in a state |D(heads)〉 cannot be taken as proof that the coin was heads up at $t=t_{1}$.

### 6.3. Only $\bar{F}$’s Record Is Preserved

Next, assume that $\bar{W}$ measures in the basis (20) and preserves $\bar{F}$’s record, while *W* erases the record of *F*. Now, in Figure 1, only the paths passing through the states |heads〉 and |tails〉 can be distinguished by consulting the existing records at the end of the experiment. The paths arriving at a final state via |tails〉|down〉 and |tails〉|up〉 at $t=t_{2}$ cannot be told apart, and, according to the rules of Section 2, their amplitudes should be added. There are eight real paths connecting the observed outcomes (see Figure 2b) and a set of eight probabilities:

(24) $$\begin{array}{cc} P^{\prime }\left(\bar{Fail},Fail,Heads\right) & =\;|A_{1}{|}^{2}=1/12,\;P^{\prime }\left(\bar{Fail},Fail,Tails\right)={\left|A_{2}+A_{3}\right|}^{2}=1/3\; \end{array}$$

$$\begin{array}{cc} P^{\prime }\left(\bar{Fail},Ok,Heads\right) & =\;|A_{4}{|}^{2}=1/12,\;P^{\prime }\left(\bar{Fail},Ok,Tails\right)={\left|A_{5}+A_{6}\right|}^{2}=0,\;\;\;\\ P^{\prime }\left(\bar{Ok},Fail,Heads\right) & =\;|A_{7}{|}^{2}=1/12,\;P^{\prime }\left(\bar{Ok},Fail,Tails\right)={\left|A_{8}+A_{9}\right|}^{2}=1/3,\;\;\\ P^{\prime }\left(\bar{Ok},Ok,Heads\right) & =\;|A_{10}{|}^{2}=1/12,\;P^{\prime }\left(\bar{Ok},Ok,Tails\right)={\left|A_{11}+A_{12}\right|}^{2}=0.\;\;\; \end{array}$$

Now, the joint probability for *W* to obtain an “Ok” and for $\bar{F}$ to see “Tails” is zero, $P^{\prime }\left(Ok,Tails\right)=P^{\prime }\left(\bar{Fail},Ok,Tails\right)+P^{\prime }\left(\bar{Ok},Ok,Tails\right)=0$ and we recover the result (13) in Section 4. Finally, having learnt that *W*’s result is “Ok”, we also know that *F* had seen “Heads”, i.e., obtained the condition (ii) of Section 4:

(25) Ok→Heads.

### 6.4. Only *F*’s Record Is Preserved

If *W* chooses the basis (21), one is unable to distinguish between the virtual paths in Figure 1, which arrive at the same final state via |heads〉|down〉 and |tails〉|down〉 at $t=t_{1}$. As before, there are eight real paths connecting the observed outcomes (see Figure 2c), and the eight new probabilities are given by:

(26) $$\begin{array}{cc} P^{\prime \prime }\left(\bar{Fail},Fail,Up\right) & =\;|A_{3}{|}^{2}=1/12,\;P^{\prime \prime }\left(\bar{Fail},Fail,Down\right)={\left|A_{1}+A_{2}\right|}^{2}=1/3,\; \end{array}$$

$$\begin{array}{cc} P^{\prime \prime }\left(\bar{Fail},Ok,Up\right) & =\;|A_{6}{|}^{2}=1/12,\;P^{\prime \prime \prime }\left(\bar{Fail},Ok,Down\right)={\left|A_{4}+A_{5}\right|}^{2}=1/3,\;\;\\ P^{\prime \prime }\left(\bar{Ok},Fail,Up\right) & =\;|A_{9}{|}^{2}=1/12,\;P^{\prime \prime \prime }\left(\bar{Ok},Fail,Down\right)={\left|A_{7}+A_{8}\right|}^{2}=0,\;\;\; \end{array}$$

(27) $$\begin{array}{cc} P^{\prime \prime }\left(\bar{Ok},Ok,Up\right) & =\;|A_{12}{|}^{2}=1/12,\;P^{\prime \prime }\left(\bar{Ok},Ok,Down\right)={\left|A_{10}+A_{11}\right|}^{2}=0.\; \end{array}$$

From this, we learn that the odds on *F* and $\bar{W}$ seeing “Down” and “$\bar{Ok}$”, respectively, are zero, $P^{\prime \prime }\left(\bar{Ok},Down\right)=P^{\prime \prime }\left(\bar{Ok},Fail,Down\right)+P^{\prime \prime }\left(\bar{Ok},Ok,Down\right)=0$, recovering the result (16). Therefore, knowing that $\bar{W}$ obtains “$\bar{Ok}$”, we also know that *F*’s result was “Up”. This is the condition (i) of Section 4:

(28) $$\bar{Ok}\to Up.$$

### 6.5. Both $\bar{F}$’s and F’s Records Are Preserved

If *W* and $\bar{W}$ measure in the bases (20) and (21), respectively, and leave $\bar{F}$’s and *F*’s records untouched, all paths can be distinguished when the experiment is finished. Each path can be endowed with a probability $|A_{i}{|}^{2}$, i=1,⋯,12 and we write down the 12 possible outcomes in a compact form as:

(29) $$\begin{array}{c} P(N,M,K,I)=1/12,\;M,K\neq Up,Heads \end{array}$$

$$\begin{array}{c} P(N,Up,Heads,I)=0,\;\;\;\;\;\;\;\; \end{array}$$

where:

(30) $$I=Heads,Tails,\;K=Up,Down,\;M=\bar{Ok},\bar{Fail},\;N=Ok,Fail.$$

The network of the real pathways connecting possible outcomes is shown in Figure 2d. The joint probability of $\bar{F}$ and *F* seeing “Heads” and “Up” vanishes since in Figure 2d no pathway connects the outcomes Heads and Up. We also have:

(31) $$\begin{array}{c} P\left(Down,Heads\right)=\sum_{M,N}P\left(N,M,Down,Heads\right)=1/3, \end{array}$$

$$\begin{array}{c} P\left(Up,Tails\right)=\sum_{M,N}P\left(N,M,Up,Tails\right)=1/3,\\ P\left(Down,Tails\right)=\sum_{M,N}P\left(N,M,Down,Tails\right)=1/3, \end{array}$$

and recover Equations (13) and (15), as well as the condition (iii) of Section 4:

(32) Up→Tails.

This is the only scenario in which the measurement records of all four agents are retained, and the joint probabilities for all outcomes exist.

## 7. Discussion and Conclusions

Consider an experiment consisting of measuring, one after another, *L* quantities, $Q^{\ell }$, taking certain discrete values $Q_{m_{\ell }}^{\ell }$. If the system under consideration is stochastic, i.e., none of the values $Q_{m_{\ell }}^{\ell }$ can be uniquely deduced from other outcomes, the experiment relies on recording the measured values and keeping the records until the last value, $Q_{m_{L}}^{L}$, is measured. Individual outcomes can be written down on a piece of paper, memorised, or encoded into any material object. In the end, the records are compared, and a result $\left\{Q_{m_{L}}^{L},\cdots Q_{m_{\ell }}^{\ell },\cdots Q_{m_{1}}^{1}\right\}$ is established. Repeating the procedure many times, one evaluates the probabilities for all possible results, $P(Q_{m_{L}}^{L}\cdots \leftarrow Q_{m_{\ell }}^{\ell }\cdots \leftarrow Q_{m_{1}}^{1})$. If a particular record, say, that of a value $Q_{m_{\ell }}^{\ell }$ is destroyed or erased in each run, the lost value cannot be recovered, and the experiment proceeds as if it was not measured at all.

Quantum systems are, by their very nature, stochastic, and all that was just stated applies also to successive quantum measurements. There is, however, one important difference. If classically one fails to record, or to keep the record of a value $Q_{m_{\ell }}^{\ell }$, the measured probability distribution, $P(Q_{m_{L}\cdots }^{L}\leftarrow Q_{m_{\ell +1}}^{\ell +1},Q_{m_{\ell -1}}^{\ell -1}\cdots \leftarrow Q_{m_{1}}^{1})$, is a marginal of the distribution obtained in a more detailed experiment,

$$P_{class}\left(Q_{m_{L}\cdots }^{L}\leftarrow Q_{m_{\ell +1}}^{\ell +1},Q_{m_{\ell -1}}^{\ell -1}\cdots \leftarrow Q_{m_{1}}^{1}\right)=\sum_{m_{\ell }}P_{class}\left(Q_{m_{L}}^{L}\cdots \leftarrow Q_{m_{\ell }}^{\ell }\cdots \leftarrow Q_{m_{1}}^{1}\right).$$

In general, this rule does not hold in the quantum case. This “only mystery of quantum mechanics” was illustrated by Feynman [15] on the simplest case of Young’s double-slit experiment, where the failure to produce a record of a slit taken by an electron, or its subsequent destruction [17], leads to the appearance of an interference pattern, otherwise absent. The same principle applies to more complex situations, such as the one considered in this paper. Quantum mechanics does not hesitate to predict probabilities in all practical situations. However, the resulting statistical ensembles depend on the retained measurement records, and are not guaranteed to be compatible.

Indeed, let us look at the three conflicting statements of Section 5. The last of them (iii), is obtained under the assumption that both $\bar{F}$ and *F* make their measurements, and their records are preserved until the end of the experiment. The other two conditions, (ii) and (i), correspond to the cases where the record of one of the Friends, $\bar{F}$ or *F*, is either erased by $\bar{W}$’s or *W*’s. Each claim is correct under the proper circumstances, and one is never in doubt as to which one is to be used. Feynman rules specify, depending on the measurements that are effectively recorded, whether one should sum over virtual paths (amplitudes) or over real paths (probabilities).

It is also easy to see what caused the apparent contradiction in Section 5. The unitarily evolved state (12) contains no information about $\bar{W}$’s and *W*’s arrangements, and must cater for all eventualities. Having stipulated that all records of *F*’s and $\bar{F}$’s measurements are to be erased by the actions of *W* and $\bar{W}$, one should go straight to Equation (19). According to Feynman’s rules, there is no contradiction. Either $\bar{W}$ and *W* measure the entire laboratories, thus, erasing $\bar{F}$’s and *F*’s records, or preserve the records by measuring only a part of a lab. One cannot have both situations at the same time without violating the rules. This would then imply that measurement outcomes would still be defined although all the corresponding material records in the Universe (including those kept in F’s brain) were erased.

In summary, quantum mechanics can be wrong in its predictions, but it is not inconsistent, at least as far as the Wigner’s Friend scenarios discussed here are concerned. It is, of course, still legitimate to question Feynman’s principle by anticipating situations in which they would fail, or where different interpretations, such as used in [1,2,3,4,5,6,7,8,9,10,11] would make verifiable alternative predictions, one would need a stronger case to make further progress in this direction.

## Appendix A. Application of Feynman’s Rules

In order to apply the principles (A–F) given in Section 2, the following steps are required:

(a) Decide which quantities are to be measured, and at what times. With each chosen quantity, associate a hermitian operator whose eigenvalues will be a possible measured outcome. The first measurement must prepare the composite in a known initial state.
(b) Define the bases needed for all measurements in the Hilbert space of a composite system which includes all subsystems interacting with each other in the course of the experiment. Connect all states, to obtain all virtual paths.
(c) The probability amplitude of a path is given by a product of the matrix elements of the evolution operators between the times of two consecutive observations.
(d) Decide which paths will interfere, depending on the degeneracies of the eigenvalues obtained in the intermediate measurements. Add up the amplitudes for the paths leading to the same final state, and take the absolute square.
(e) Sum the probabilities obtained in (d) over the degeneracies of the last eigenvalue.

It is important to bear in mind that different sets of measurements can produce different (incompatible) statistical ensembles, even if they are created on the same system, and even in the case when the measured operators commute, but have a different set of degenerate eigenvalues (see, e.g., [18]).

Finally, the probabilities one calculates ought to be the likelihoods of the outcomes experienced by all Observers taking part in the experiment. Such experiences may be gained by registering the conditions of the devices (probes) accessible to an Observer at the end of experiment, or by consulting an Observer’s memory, which carries a record of a previously registered outcome [17,19].

## Appendix B. A Double-Slit Example

In [15], Feynman illustrated their basic principles of quantum motion on a double-slit experiment, where a photon is scattered by an electron into one of two distinguishable states, depending on the slit chosen by the electron. The presence of such a photon allows, in principle, to determine which slit was used, and the interference pattern on the screen is destroyed even if the photon is *never observed*.

The simplest double-slit problem can be conceived by considering a two-level system, *S*, and two probes, *D* and $\bar{D}$. The probes will have no own dynamics, and move only as a result of coupling with other parts of the joint system. At $t=t_{0}$, the three are prepared in a state $|\Psi_{0}\rangle =|\bar{D}\left(0\right)\rangle ⊗\left|D\left(0\right)\rangle ⊗\right|s_{0}\rangle$, and at $t_{1}>t_{0}$ and at $t_{2}>t_{1}$ the two probes coupled to the system according to (|s〉 is an arbitrary system’s state, and |up〉, down〉 and |fail〉, ok〉 are two orthogonal bases in its Hilbert space):

(A1) $$\begin{array}{c} {\hat{U}}^{D+S}\left(t_{1}\right)|D\left(0\right)\rangle ⊗|s\rangle =\langle up|s\rangle |D\left(up\right)\rangle ⊗|up\rangle +\langle down|s\rangle |D\left(down\right)\rangle ⊗|down\rangle , \end{array}$$

$$\begin{array}{c} {\hat{U}}^{\bar{D}+S}\left(t_{2}\right)|\bar{D}\left(0\right)\rangle ⊗|s\rangle =\langle fail|s\rangle |\bar{D}\left(fail\right)\rangle ⊗|fail\rangle +\langle ok|s\rangle |\bar{D}\left(ok\right)\rangle ⊗|ok\rangle , \end{array}$$

where:

(A2) $$\begin{array}{c} |fail\rangle =\alpha |up\rangle +\beta |down\rangle ,\;|ok\rangle =\gamma |up\rangle +\delta |down\rangle . \end{array}$$

The unitary evolution of $|\Psi_{0}\rangle$ to a time just after $t_{2}$ yields:

(A3) $$\begin{array}{c} |\Psi \left(t\right)\rangle \equiv u^{\bar{D}+S}\left(t_{2}\right){\hat{U}}^{S}\left(t_{2},t_{1}\right){\hat{U}}^{D+S}\left(t_{1}\right){\hat{U}}^{S}\left(t_{1},t_{0}\right)|\bar{D}\left(0\right)\rangle ⊗\left|D\left(0\right)\rangle ⊗\right|s_{0}\rangle \; \end{array}$$

$$\begin{array}{c} =\sum_{i,j}A^{S}\left(j\leftarrow i\leftarrow s_{0}\right)|\bar{D}\left(j\right)\rangle ⊗\left|D\left(i\right)\rangle ⊗\right|j\rangle ,\;\;\;\;\;\;\;\;\\ A^{S}\left(j\leftarrow i\leftarrow s_{0}\right)\equiv \langle j|{\hat{U}}^{S}\left(t_{2},t-1\right)\left|i\rangle \langle i\right|{\hat{U}}^{S}\left(t_{1},t_{0}\right)|s_{0}\rangle ,\;i=up,down,\;j=fail,ok, \end{array}$$

where $A^{S}\left(j\leftarrow i\leftarrow s_{0}\right)$ is the amplitude of a system’s virtual path $\left\{j\leftarrow i\leftarrow s_{0}\right\}$, and ${\hat{U}}^{S}$ is the system’s evolution operator. The joint probability of *F* and *W* seeing the outcomes *i* and *j*:

(A4) $$\begin{array}{c} P\left(i,j\right)\equiv \langle \Psi \left(t\right)|\bar{D}\left(j\right)\rangle \langle \bar{D}\left(j\right)|⊗|D\left(i\right)\rangle \langle D\left(i\right)|\Psi \left(t\right)\rangle =\;|A^{S}\left(j\leftarrow i\leftarrow s_{0}\right){|}^{2}, \end{array}$$

and, in particular, the odds of *W* seeing an outcome Ok are:

(A5) $$\begin{array}{c} P\left(ok\right)\equiv P\left(ok,up\right)+P\left(ok,down\right)=\;|A^{S}\left(ok\leftarrow up\leftarrow s_{0}\right){|}^{2}+{\left|A^{S}\left(ok\leftarrow down\leftarrow s_{0}\right)\right|}^{2}\; \end{array}$$

In a different scenario, *F* may decide to measure, so their probe, not coupled to the system, continues in its initial state |D(0)〉. Now, the unitary evolution yields become:

(A6) $$\begin{array}{c} |\Psi^{\prime }\left(t\right)\rangle \equiv u^{D+S}\left(t_{2}\right){\hat{U}}^{S}\left(t_{2},t_{0}\right)|\bar{D}\left(0\right)\rangle ⊗\left|D\left(0\right)\rangle ⊗\right|s_{0}\rangle =\;\;\;\; \end{array}$$

$$\begin{array}{c} \left[A^{S}\left(fail\leftarrow up\leftarrow s_{0}\right)+A^{S}\left(fail\leftarrow down\leftarrow s_{0}\right)\right]|\bar{D}\left(fail\right)\rangle ⊗\left|fail\rangle ⊗\right|D\left(0\right)\rangle \\ +\left[A^{S}\left(ok\leftarrow up\leftarrow s_{0}\right)+A^{S}\left(ok\leftarrow down\leftarrow s_{0}\right)\right]|\bar{D}\left(ok\right)\rangle ⊗\left|ok\rangle ⊗\right|D\left(0\right)\rangle ,\; \end{array}$$

We, therefore, have:

(A7) $$\begin{array}{c} P^{\prime }\left(ok\right)\equiv \langle \Psi \left(t\right)|\bar{D}\left(ok\right)\rangle \langle \bar{D}\left(ok\right)|\Psi \left(t\right)\rangle =\;|A^{S}\left(ok\leftarrow up\leftarrow s_{0}\right)+A^{S}\left(ok\leftarrow down\leftarrow s_{0}\right){|}^{2},\; \end{array}$$

and need not examine the first probe, decoupled from the system of interest.

Comparing Equations (A4) and (A6), one makes two useful observations. Firstly, if only von Neumann couplings are applied, the probability of interest can be expressed via the probability amplitudes evaluated for the studied system in the absence of the probes. This was first noted by von Neumann [20].

Secondly, the probabilities can be constructed according to Feynman’s rules applied to the studied system only, whose virtual paths interfere if *D* is not engaged, but become exclusive “real” alternatives if *D* is coupled, and the paths can be distinguished.

One thing that can be added to this simple narrative is this [17]: The Observer may set up the second probe to measure a composite {system+D}. The simplest double-slit problem can be conceived by considering a two-level system, *S*, and two probes, *D* and $\bar{D}$ in a basis (the evolution will occur in a two dimensional sub-space of the four-dimensional Hilbert space, and we will not bother to specify all four basis states):

(A8) $$\begin{array}{c} |Fail\rangle =\alpha |D(up)\rangle ⊗|up\rangle +\beta |D(down)\rangle ⊗|down\rangle , \end{array}$$

$$\begin{array}{c} |Ok\rangle =\gamma |D(up)\rangle ⊗|up\rangle +\delta |D(down)\rangle ⊗|down\rangle .\; \end{array}$$

The new coupling of *W*’s probe, therefore, is (|Φ〉 is any state of the composite in the sub-space spanned by |Fail〉 and |Ok〉):

(A9) $$\begin{array}{c} {\hat{U}}^{Pr^{\prime }+Pr+S}\left(t_{2}\right)|\bar{D}\left(0\right)\rangle ⊗|\Phi \rangle =\langle Fail|\Phi \rangle |\bar{D}\left(Fail\right)\rangle ⊗|Fail\rangle +\langle Ok|\Phi \rangle |\bar{D}\left(Ok\right)\rangle ⊗|Ok\rangle ,\; \end{array}$$

and for the unitarily evolved state, just after $t_{2}$, we find:

(A10) $$\begin{array}{c} |\Psi^{\prime \prime }\left(t\right)\rangle \equiv {\hat{U}}^{Pr^{\prime }+Pr+S}\left(t_{2}\right){\hat{U}}^{S}\left(t_{2},t_{1}\right){\hat{U}}^{Pr+S}\left(t_{1}\right){\hat{U}}^{S}\left(t_{1},t_{0}\right)|\bar{D}\left(0\right)\rangle ⊗\left|D\left(0\right)\rangle ⊗\right|s_{0}\rangle =\;\; \end{array}$$

$$\begin{array}{c} \left[A^{S}\left(fail\leftarrow up\leftarrow s_{0}\right)+A^{S}\left(fail\leftarrow down\leftarrow s_{0}\right)\right]\left|\bar{D}\left(Fail\right)\rangle ⊗\right|Fail\rangle \;\;\;\;\\ +\left[A^{S}\left(ok\leftarrow up\leftarrow s_{0}\right)+A^{S}\left(ok\leftarrow down\leftarrow s_{0}\right)\right]\left|\bar{D}\left(Ok\right)\rangle ⊗\right|Ok\rangle ,\;\;\;\;\; \end{array}$$

so that:

(A11) $$\begin{array}{c} P^{\prime \prime }\left(Ok\right)\equiv =\langle \Psi^{\prime \prime }\left(t\right)|\bar{D}\left(Ok\right)\rangle \langle \bar{D}\left(Ok\right)\left|\right|\Psi^{\prime \prime }\left(t\right)\rangle =\;\;\; \end{array}$$

$$\begin{array}{c} |A^{S}\left(ok\leftarrow up\leftarrow s_{0}\right)+A^{S}\left(ok\leftarrow down\leftarrow s_{0}\right){|}^{2}=P^{\prime }\left(ok\right). \end{array}$$

As before, the probabilities of interest can be obtained by manipulating the system’s path amplitudes, $A^{S}$, and the result still agrees with Feynman’s rules of Section 6.1. The application of the second probe (cf. (A9)) becomes *erased*, or if one prefers *destroyed*, the record carried by the first probe, the system’s paths passing through the states |up〉 or |down〉 at $t=t_{1}$ cannot be distinguished, and their amplitudes must be added.

Note that one can still check whether the first probe is in a state |D(up)〉 at some $t_{3}>t_{2}$, e.g., just after $t_{2}$, so that ${\hat{U}}^{S}\left(t_{3},t_{2}\right)=1$. In this case, using Feynman’s rules (in the Hilbert space of {S+D}) and evaluating the matrix elements, one easily finds:

(A12) $$\begin{array}{c} P^{\prime \prime \prime }\left(up,Ok\right)=\sum_{i=up,down}{\left|A^{S+D}\left(up⊗D\left(i\right)\leftarrow Ok\leftarrow D\left(0\right)⊗s_{0}\right)\right|}^{2} \end{array}$$

$$\begin{array}{c} =\;|A^{S+D}\left(up⊗D\left(up\right)\leftarrow Ok\leftarrow D\left(0\right)⊗s_{0}\right){|}^{2}={\left|A^{S}\left(up\leftarrow ok\leftarrow up\leftarrow s_{0}\right)\right|}^{2}\\ ={\left|\gamma \right|}^{2}{\left|A^{S}\left(ok\leftarrow up\leftarrow s_{0}\right)+A^{S}\left(ok\leftarrow down\leftarrow s_{0}\right)\right|}^{2}= \end{array}$$

and:

(A13) $$\begin{array}{c} P^{\prime \prime \prime }\left(up,Fail\right)={\left|\alpha \right|}^{2}{\left|A^{S}\left(ok\leftarrow up\leftarrow s_{0}\right)+A^{S}\left(ok\leftarrow down\leftarrow s_{0}\right)\right|}^{2}. \end{array}$$

Finally, since $\langle j^{\prime }|j\rangle =\delta_{j^{\prime }j}$, $j,j^{\prime }=Fail,Ok$, we have $\gamma =\beta^{*}$, so that:

(A14) $$\begin{array}{c} P^{\prime \prime \prime }\left(up\right)=P^{\prime \prime \prime }\left(up,Ok\right)+P^{\prime \prime \prime }\left(up,Fail\right)={\left|A^{S}\left(ok\leftarrow up\leftarrow s_{0}\right)+A^{S}\left(ok\leftarrow down\leftarrow s_{0}\right)\right|}^{2},\;\;\; \end{array}$$

and, as expected, the information about the system’s state at $t_{1}$ is lost to interference.
